# Acute-on-chronic liver failure: far to go—a review

**DOI:** 10.1186/s13054-023-04540-4

**Published:** 2023-07-01

**Authors:** Jinjin Luo, Jiaqi Li, Peng Li, Xi Liang, Hozeifa Mohamed Hassan, Richard Moreau, Jun Li

**Affiliations:** 1 State Key Laboratory for Diagnosis and Treatment of Infectious Diseases, National Clinical Research Center for Infectious Diseases, National Medical Center for Infectious Diseases, The First Affiliated Hospital, Zhejiang University School of Medicine, 79 Qingchun Rd., Hangzhou, 310003 China; 2 Department of Gastroenterology, Zhejiang Provincial People’s Hospital, People’s Hospital Affiliated of Hangzhou Medical College, Hangzhou, China; 3 Precision Medicine Center, Taizhou Central Hospital (Taizhou University Hospital), Taizhou, China; 4 European Foundation for the Study of Chronic Liver Failure (EF CLIF), Barcelona, Spain; 5 Centre de Recherche Surl’Inflammation (CRI), Institut National de La Santé Et de La Recherche Médicale (INSERM) & Université Paris-Cité, Paris, France; 6 Service d’Hépatologie, Assistance Publique–Hôpitaux de Paris (APHP), Hôpital Beaujon, Clichy, France

**Keywords:** Acute-on-chronic liver failure, Organ failure, Prognosis, Systemic inflammatory response, Immune-metabolism disorder, Clinical management

## Abstract

Acute-on-chronic liver failure (ACLF) has been recognized as a severe clinical syndrome based on the acute deterioration of chronic liver disease and is characterized by organ failure and high short-term mortality. Heterogeneous definitions and diagnostic criteria for the clinical condition have been proposed in different geographic regions due to the differences in aetiologies and precipitating events. Several predictive and prognostic scores have been developed and validated to guide clinical management. The specific pathophysiology of ACLF remains uncertain and is mainly associated with an intense systemic inflammatory response and immune-metabolism disorder based on current evidence. For ACLF patients, standardization of the treatment paradigm is required for different disease stages that may provide targeted treatment strategies for individual needs.

## Introduction

Acute-on-chronic liver failure (ACLF) is a life-threatening clinical syndrome that develops in patients with acute deterioration of chronic liver disease [1]. In Western countries, alcohol-related cirrhosis is the most common cause of chronic liver disease, with bacterial infections being the most important precipitant of ACLF. In contrast, in the Asia–Pacific region, hepatitis B virus (HBV) infection is the most common aetiology of liver disease, and HBV reactivation is the most frequent ACLF trigger/hepatic event [2]. The definitions of ACLF currently vary worldwide, but despite these differences, patients with ACLF uniformly have a high risk of short-term mortality with multiorgan failure [1]. New results have been published suggesting the existence of a unique pathophysiology of patients with HBV-ACLF [3]. Meanwhile, the current evolution of ACLF clinical management involves the emergence of potential therapeutic strategies. This review provides an updated summary of the diagnostic criteria and prognostic scores proposed by different scientific societies, the underlying pathophysiology of ACLF and its treatment.

## Definitions

In the past decade, several definitions of ACLF have been proposed by international consortia (summarized in Table 1). The main controversies involve the type of precipitating events (intrahepatic or extrahepatic), the stage of underlying chronic liver disease leading to ACLF (chronic hepatitis or cirrhosis) and whether the definition should include extrahepatic organ failures (OFs).

**Table 1:** Definitions, diagnostic criteria and stratification of ACLF proposed by each of the four major international scientific consortia

Characteristics	European Association for the Study of the Liver-Chronic Liver Failure (EASL-CLIF) Consortium	Chinese Group on the Study of Severe Hepatitis B (COSSH)	Asian Pacific Association for the Study of the Liver (APASL) ACLF Research Consortium (AARC)	North American Consortium for the Study of End-stage Liver Disease (NACSELD)
Study type	Multicentre, prospective, observational study	Multicentre, prospective, observational study	Expert consensus report	Multicentre, prospective, observational study
Main aetiology	Alcohol, HCV	HBV	HBV	Alcohol
Precipitating events	Intrahepatic (alcoholic hepatitis), extrahepatic (infection, variceal haemorrhage), or both	Intrahepatic (HBV reactivation), extrahepatic (bacterial infection) or both	Intrahepatic	Intrahepatic, extrahepatic or both
Population	Compensated and decompensated cirrhosis	HBV-related chronic liver disease	Chronic liver disease Compensated cirrhosis	Compensated and decompensated cirrhosis
Criteria of Organ failure	Liver: total bilirubin ≥ 12 mg/dL; Kidney: creatinine ≥ 2 mg/dL or use of RRT; Coagulation: INR ≥ 2.5; Brain: West Haven grade 3–4 HE or use of mechanical ventilation due to HE; Circulation: Use of vasopressors; Respiration: PaO2/FiO2 ≤ 200 or SpO2/FiO2 ≤ 214, or use of mechanical ventilation not due to HE	Liver: Total bilirubin ≥ 12 mg/dL; Kidney: Creatinine ≥ 2 mg/dL or use of RRT; Coagulation: INR ≥ 2.5; Brain: West Haven grade 3–4 HE or use of mechanical ventilation due to HE; Circulation: Use of vasopressors; Respiration: PaO2/FiO2 ≤ 200 or SpO2/FiO2 ≤ 214, or use of mechanical ventilation not due to HE	Liver: Total bilirubin ≥ 5 mg/dL; Brain: clinical HE	Kidney: Use of dialysis or other form of RRT; Brain: HE Grade 3–4 in West Haven classification; Circulation: MAP < 60 mmHg or reduction of 40 mmHg in SBP from baseline, in spite of fluid resuscitation and adequate cardiac output; Respiration: Use of mechanical ventilation
Criteria for the presence of ACLF and ACLF stratification	ACLF is divided into 3 grades of increasing severity Grade 1 includes 3 subgroups: (1) single kidney failure; (2) single liver, coagulation, circulatory or respiratory failure with either kidney dysfunction, brain dysfunction, or both; (3) single brain failure and kidney dysfunction; Grade 2: 2 organ failures; Grade 3: 3 or more organ failures	ACLF is divided into 3 grades of increasing severity Grade 1 includes 4 subgroups: (1) single kidney failure; (2) single liver failure and either INR ≥ 1.5, kidney dysfunction, brain dysfunction, or any combination of these alterations; (3) single coagulation, circulatory or respiratory failure with either kidney dysfunction, brain dysfunction, or both; (4) brain failure alone plus kidney dysfunction; Grade 2: 2 organ failures; Grade 3: 3 or more organ failures	Acute hepatic insult manifesting as jaundice (total bilirubin levels of 5 mg/dl or more) and coagulopathy (INR ≥ 1.5, or prothrombin activity < 40%) complicated within 4 weeks by clinical ascites, HE, or both The severity of ACLF is assessed using the AARC score Grade 1: 5–7 scores, Grade 2: 8–10 scores, Grade 3: 11–15 scores	Patients are stratified according to the number of organ failures 2, 3, or all 4 organ failures
Prevalence of ACLF grade	Grade 1: 49% Grade 2: 35% Grade 3: 16%	Grade 1: 61% Grade 2: 33% Grade 3: 6%	/	2 Organ failures: 43% 3 Organ failures: 41% 4 Organ failures: 16%
Main organ failures	Kidney, Liver	Liver, Coagulation	Liver, Coagulation	Brain
Short-term mortality rate of ACLF	By 28 days: Grade 1: 22% Grade 2: 32% Grade 3: 77%	By 28 days: Grade 1: 23% Grade 2: 61% Grade 3: 93%	By 28 days: Grade 1: 13% Grade 2: 45% Grade 3: 86%	By 30 days: 2 Organ failures: 49% 3 Organ failures: 64% 4 Organ failures: 77%

*ACLF* acute-on-chronic liver failure; *FiO*_*2*_ fraction of inspired oxygen; *HBV* hepatitis B virus; *HCV* hepatitis C virus; *HE* hepatic encephalopathy; *INR* international normalized ratio; *MAP* mean arterial pressure; *PaO*_*2*_ partial pressure of arterial oxygen; *RRT* renal replacement therapy; *SBP* systolic blood pressure; *SpO*_*2*_ pulse oximetric saturation

### European Association for the Study of the Liver-Chronic Liver Failure (EASL-CLIF) ACLF

To define ACLF as a distinct syndrome, a multicentre, prospective and observational (chronic liver failure consortium acute-on-chronic liver failure in cirrhosis) CANONIC study, involving 1343 patients nonelectively hospitalized for acute decompensation of cirrhosis with or without prior decompensation, was performed in Europe to determine the characteristics of patients with OFs; a ≥ 15% mortality rate at 28 days was observed [4]. The EASL-CLIF ACLF definition includes both intra- and extra-hepatic precipitating events and emphasizes the importance of extrahepatic OFs rather than liver failure alone. The diagnosis of organ failure is based on the CLIF-C OF scoring system as a modified sequential organ failure assessment (SOFA) score, which assesses six organ systems (liver, kidney, brain, coagulation, circulation and respiration) [5]. The criteria comprise three grades with increasing severity. Patients with ACLF have a single kidney failure (ACLF grade 1/ACLF-1); a single nonkidney organ failure if it is associated with kidney or brain dysfunction (ACLF-1); or more than 2 OFs (ACLF-2 or 3) on the basis of the type and number of OF(s).

### Chinese Group on the Study of Severe Hepatitis B (COSSH) ACLF

To clarify the characteristics of ACLF in the HBV population, a definition was proposed by the prospective and observational COSSH study based on data from 1322 patients nonelectively hospitalized with cirrhosis or severe liver injury due to chronic hepatitis B. The COSSH study found that patients with HBV-ACLF had significantly worse clinical characteristics, and the short-term mortality of patients with noncirrhotic HBV-ACLF was significantly higher than that of patients with non-HBV-ACLF. Regardless of the presence of cirrhosis, patients with a total bilirubin (TB) ≥ 12 mg/dL and an international normalized ratio (INR) ≥ 1.5 had a higher short-term mortality [6]. Three ACLF grades were also proposed to more easily categorize patients with ACLF based on criteria that were similar to the European definition; however, patients with single liver failure and an INR of ≥ 1.5 were additionally diagnosed with ACLF grade 1. The COSSH-ACLF criteria exhibited higher diagnostic sensitivity and prognostic accuracy and bridged the gap in the EASL-CLIF criteria for HBV-ACLF diagnosis.

### Asian Pacific Association for the Study of the Liver (APASL) ACLF

Based on expert opinion, a definition was published by the APASL ACLF Research Consortium (AARC) in 2009. ACLF was defined as a syndrome of acute liver function damage on the basis of known or unknown chronic liver disease (TB ≥ 5 mg/dl and INR ≥ 1.5 or prothrombin activity ≤ 40%) accompanied by ascites and/or hepatic encephalopathy within 4 weeks. The features of “high 28-day fatality rate” and “reversibility of the ACLF syndrome” were added as parts of the definition in subsequent updates in 2014 and 2019 [7–9]. The definition mainly includes patients with compensated liver disease and excludes patients with prior decompensation. Primary intrahepatic insults are considered the only precipitating events; extrahepatic insults, such as bacterial infection, are considered to be complications but not precipitating events of ACLF. Liver failure is essential in diagnosing ACLF, whereas extrahepatic OFs are not required to make the diagnosis and are considered to be manifestations that are concurrent with the development of ACLF.

### North American Consortium for the Study of End-stage Liver Disease (NACSELD) ACLF

The NACSELD proposed a definition of ACLF in 2014, based on the analysis of the NACSELD database, which included 507 patients with acutely decompensated cirrhosis with infection [10]. Four organ systems (circulation, respiration, kidney, brain) were assessed, and the presence of ≥ 2 OFs was defined as ACLF. The definition did not include changes in liver function and coagulation, which are the typical pathophysiological manifestations of ACLF.

## Prediction and prognosis

The accuracy and sensitivity of predictive and prognostic scores are important to make decisions regarding intensive care strategies and predict outcomes in patients with ACLF. The populations, variables, formulas and applications of commonly used scores developed by the abovementioned consortia are summarized in Table 2 [4–6, 11–15].

**Table 2:** Predictive and prognostic scores developed by different consortia

Scores	Population	Variable	Formula	Application
*Prognostic scores*				
CLIF-C ACLFs	Acutely decompensated cirrhosis	CLIF‐OFs (TB, creatinine, use of RRT, HE, endotracheal intubation for HE, INR, MAP, Use of vasopressors, PaO2, SpO2, FiO2), age, WBC	10 × [0.33 × CLIF-OFs) + 0.04 × age + 0.63 × ln (WBC, 10^9^/L) − 2]	Individual estimates of mortality
COSSH-ACLFs	Acute deterioration of HBV-related chronic liver disease	HBV-SOFAs (creatinine, HE, MAP, Use of vasopressors, PaO2, SpO2, FiO2), INR, age, TB	0.741 × INR + 0.523 × HBV-SOFAs + 0.026 × age + 0.003 × TB (μmol/L)	/
COSSH-ACLF IIs	Acute deterioration of HBV-related chronic liver disease	INR, HE, neutrophil, TB, serum urea, age	1.649 × ln(INR) + 0.457 × HE score (0/I-II/III-IV: 1/2/3 points) + 0.425 × ln(neutrophil, 10^9^/L) + 0.396 × ln(TB, umol/L) + 0.576 × ln(serum urea, mmol/L) + 0.033 × age	Individual estimates of mortality; Risk stratification: 28-/90-day Mortality: Low-risk (< 7.4): 8%/19%; Intermediate-risk (7.4–8.4): 50%/66%; High-risk (≥ 8.4): 76%/88%
AARC-ACLFs	Acute deterioration of chronic liver disease	TB, creatinine, serum lactate, INR, HE	TB (< 15/15–25/ > 25 mg/dl; 1/2/3 points) + HE (0/I-II/III-IV; 1/2/3 points) + INR (< 1.8/1.8–2.5/ > 2.5; 1/2/3 points) + Lactate (< 1.5/1.5–2.5/ > 2.5 mmol/L;1/2/3 points) + creatinine (< 0.7/0.7–1.5/ > 1.5 mg/dl; 1/2/3 points)	Risk stratification: 28-day Mortality: Grade-I (5–7): 13%; Grade-II (8–10): 45%; Grade-III (11–15): 86%
*Predictive scores*				
CLIF-C ACLF-Ds	Acutely decompensated cirrhosis without ACLF	Age, ascites, WBC, albumin, TB, creatinine	0.03 × Age + 0.45 × Ascites + 0.26 × ln(WBC)–(0.37 × Albumin) + 0.57 × ln(TB) + 1.72 × ln(Creatinine) + 3 × 10	Predicting the onset of ACLF, but showed a similar accuracy to other scores
COSSH-onset- ACLFs	Acute deterioration of HBV-related chronic liver disease without ACLF	ALT, TB, INR, serum ferritin	0.101 × ln(ALT) + 0.819 × ln(TB) + 2.820 × ln(INR) + 0.016 × ln(ferritin)	Predicting the onset of HBV-ACLF at 7/14/28 days after admission; Risk stratification for progression to ACLF at 7/14/28 days: Low-risk (< 6.3): 2.5%/3.2%/3.7%; High-risk (≥ 6.3): 42.6%/49.2%/50%

*ACLF* acute-on-chronic liver failure; *AARC* Asian Pacific Association for the Study of the Liver ACLF Research Consortium; *HBV* hepatitis B virus; *CLIF-C* Chronic Liver Failure-Consortium; *COSSH* Chinese Group on the Study of Severe Hepatitis B; *HE* hepatic encephalopathy; *INR* international normalized ratio; *MAP* mean arterial pressure; NACSELD; North American Consortium for the Study of End-stage Liver Disease; *OF* organ failure; *RRT* renal replacement therapy; *SOFA* sequential organ failure assessment; *TB* total bilirubin; *WBC* white blood cell

## Pathophysiology

### Systemic inflammation

**Fig. 1:**
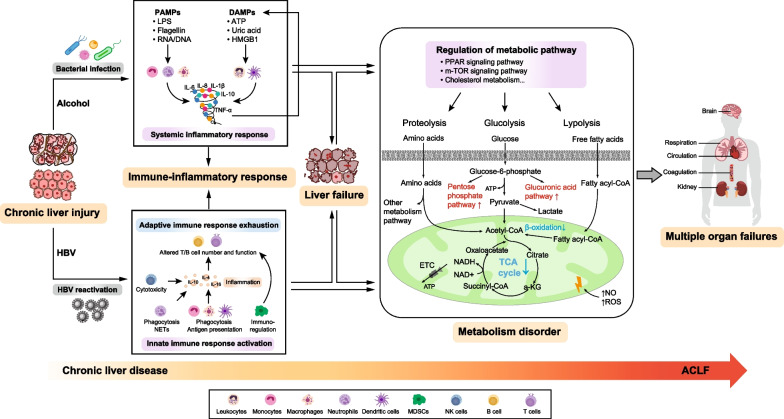
Pathophysiology of ACLF. Chronic liver diseases related to alcohol and HBV under precipitating events (bacterial infection and HBV reactivation) induce a dramatic immune-inflammatory response and metabolism disorder and eventually develop into multiple organ failures. The systemic inflammatory response plays an important role in the development of ALD-ACLF. The released PAMPs and DAMPs from bacteria and necrotic cells activate immune cells and result in the increased release of inflammatory mediators and even a cytokine storm. Activation of the innate-immune system and exhaustion of the adaptive immune system are the core mechanisms of HBV-ACLF. Alterations in metabolic pathways regulate glycolysis, proteolysis and lipolysis in the context of immune-inflammatory disorder and liver failure. Glucose is used to rapidly produce ATP through glycolysis and enters the pentose phosphate pathway and glucuronic acid pathway, whereas mitochondrial oxidative phosphorylation is suppressed; mitochondrial β-oxidation of fatty acids is inhibited; and increased generation and accumulation of amino acids are metabolized through specific metabolic pathways. ACLF, acute-on-chronic liver failure; ATP, adenosine triphosphate; HBV, hepatitis B virus; CoA, coenzyme A; DAMPs, damage-associated molecular patterns; ETC, electron transport chain; HMGB1, high mobility group box 1; IL, interleukin; LPS, lipopolysaccharide; MDSC, myeloid-derived suppressor cell; m-TOR, mammalian target of rapamycin; NETs, neutrophil extracellular traps; NO, nitric oxide; PAMPs, pathogen-associated molecular patterns; PPAR, peroxisome proliferator-activated receptors; PRRs, pattern-recognition receptors; ROS, reactive oxygen species; TCA, tricarboxylic acid; TNF-α, tumour necrosis factor-α

Studies have indicated that the intense systemic inflammatory response is the main cause of acute deterioration that leads to ACLF in patients with alcohol-related liver disease (ALD) and in those with chronic hepatitis C [16, 17]. ALD-ACLF patients have higher levels of inflammatory response indicators, such as the WBC count and C-reactive protein, which are closely related to prognosis. The levels of proinflammatory factors such as interleukin (IL)-6 and IL-8 and anti-inflammatory factors such as IL-10 in the blood are also significantly increased in these patients [4, 5, 16, 18]. Systemic inflammation can be induced by the presence of pathogen-associated molecular patterns (PAMPs) and damage-associated molecular patterns (DAMPs) [19, 20]. Bacterial infection, the primary precipitant of ACLF, can cause high levels of circulating PAMPs, which can be recognized by pattern-recognition receptors (PRRs). PRR engagement can drive intracellular signalling cascades (such as JAK2/STAT1, NF-κβ, etc.), ultimately leading to the transcription and synthesis of inflammatory mediators, an imbalance in anti-inflammatory and proinflammatory regulation, and the production of a cytokine storm [20]. Systemic inflammation can also occur in the absence of infection, a process known as sterile inflammation. In this case, the release of numerous DAMPs from dying or damaged hepatocytes (in the context of alcohol-related hepatitis or HBV activity) activates specific PRRs, triggering the release of inflammatory cytokines, which leads to an inflammatory response and ultimately ACLF (Fig. 1) [21, 22].

### Immune-metabolism disorder

ACLF is often associated with immunodeficiency [23, 24]. Liver cirrhosis, in which liver fibres and fibrous septa replace the normal endothelial reticulum structure of the liver, leads to a decrease in the Kupffer cell number and a reduction in innate-immune protein molecules and PRRs [24], which greatly increases the risk of bacterial infection, systemic inflammatory response, and sepsis. In addition, studies involving HBV-ACLF patients showed that during chronic HBV infection, the number of myeloid dendritic cells and plasmacytoid dendritic cells in the host does not change significantly, but their functions are significantly altered compared with those in healthy controls [25–28]. HBV proteins, such as hepatitis B e antigen, can affect the ability of Kupffer cells to express Toll-like receptors, inhibit the proliferation of specific T lymphocytes and the secretion of interferon-γ and IL-10 [29]. A large number of mutations in HBV surface antigen-encoding genes were found in HBV-ACLF patients, which are associated with HBV-immune escape [30]. Circulating neutrophil and monocyte counts were higher while lymphocyte counts were lower in ACLF patients than in patients without ACLF [31, 32]. Neutrophils in patients with decompensated cirrhosis and ACLF displayed impaired phagocytosis, reactive oxygen species production, and bactericidal activity but an increased capacity to form neutrophil extracellular traps [33–35]. The function of the monocyte-macrophage system is significantly inhibited in ACLF patients, with a decrease in proinflammatory factor secretion, killing bacteria, oxidative burst, and phagocytosis [36, 37]. During the progression of HBV-related liver diseases, the percentage of CD163^+^CD206^+^ macrophages increases, and the macrophage polarization gradually changes from classically activated to alternatively activated [38]. Myeloid-derived suppressor cells are expanded in patients with ACLF and attenuate antimicrobial innate-immune responses by suppressing T cell function [39, 40]. Studies have also shown that HBV reactivation can lead to a significant increase in HBV-specific CD4^+^ and CD8^+^ T lymphocytes and cause liver damage [41, 42]. The human leukocyte antigen class II-restricted CD4^+^ T cell pathway plays an important role in the immunopathogenesis of HBV-ACLF [43]. During the evolving disease course from chronic hepatitis B or liver cirrhosis to ACLF, significantly increased expression of interferon-related, monocyte-related, neutrophil-related and dendritic cell-related gene modules, and significantly decreased expression of T cell-related, B cell-related and natural killer cell-related gene modules were observed. HBV reactivation causes immune dysregulation, including activation of the innate-immune system and exhaustion of the adaptive immune system, which are the core mechanisms of HBV-ACLF (Fig. 1) [3].

The liver is the main metabolic organ that regulates the homeostasis of lipids and cholesterol, processes amino acids, and stores glucose. Recent advances indicating prevailing metabolic alterations in the disease progression of ACLF have led to new directions in our understanding of the pathophysiology of advanced liver disease. Metabolic dysregulations, such as altered serum lysophosphatidylcholine and high-density lipoprotein cholesterol levels, are associated with increased severity and mortality of decompensated liver disease that reflect hepatocyte death [44–46]. Prominent metabolic pathway alterations (including lipid metabolism, autophagy and oxygen homoeostasis) across all stages of ACLF development were observed, which highlighted the importance of immune-metabolism disorder as a potential mechanism of ACLF [3]. Meanwhile, studies using high-throughput blood metabolomics in ACLF revealed profound alterations in major metabolic pathways; in particular, a significant decrease in mitochondrial fatty acid β-oxidation leads to decreased oxidative phosphorylation and ATP production [47, 48]. During this process, blood amino acids, along with glucose, fuel the synthesis of protein and nucleotide in the activated innate-immune system, which may contribute to the deterioration of the disease [48, 49]. Moreover, mitochondrial dysfunction governs immunometabolism in leukocytes from patients with acute decompensation of cirrhosis and ACLF, and bioenergetic failure is an emerging factor in the pathophysiology (Fig. 1) [50]. Prognostic scores based on metabolites reflecting systemic inflammation, mitochondrial dysfunction and sympathetic system activation also showed good accuracy in short-term mortality assessments [51].

## Clinical treatment

### Treatment principle

**Fig. 2:**
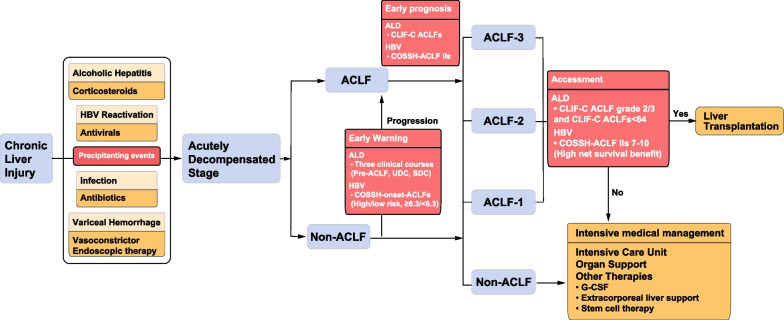
Schematic diagram showing a paradigm for ACLF management. In the chronic liver disease stage, standardization of management protocols for the treatment of precipitating events is needed to close the “switch” of acute deterioration. Once patients develop to the acutely decompensated stage, different populations could be diagnosed using different criteria depending on the phenotype specificity of ACLF. Non-ACLF patients should be predicted the risk of progression to ACLF to prevent the onset of ACLF with intense intervention, while ACLF patients should be assessed for different prognostic stratifications by scores. Patients with ACLF who can derive a high survival benefit from LT by evaluation should be prioritized for liver transplantation, making efficient use of the limited donor organs and reducing the risk of futile transplantation. ACLF, acute-on-chronic liver failure; ALD, alcohol-related liver disease; HBV, hepatitis B virus; CLIF-C ACLFs, chronic liver failure consortium ACLF score; COSSH, Chinese Group on the Study of Severe Hepatitis B; COSSH- ACLF IIs, COSSH- ACLF II score; COSSH-onset-ACLFs, COSSH-onset-ACLF score; G-CSF, granulocyte colony-stimulating; SDC, stable decompensated cirrhosis; UDC, unstable decompensated cirrhosis

Patients with ACLF should be managed in an intensive care unit by a team with expertise in critical care and liver transplantation. The main principles of treatment are to remove the precipitating cause, support failing organ(s), and perform liver transplantation in selected patients (Fig. 2). General management includes the rapid restoration of metabolic and haemodynamic stability and provision of nutritional support and agents to protect hepatocytes and promote regeneration. Vital signs and organ function should be monitored frequently, and early organ-specific supportive care should be provided with care overseen by experts in the management of liver failure [52–54].

### Treating acute precipitants

#### Hepatitis B virus reactivation

The most common precipitating disorder of patients with HBV-ACLF is HBV reactivation (more than 60% of cases) [6, 55]. Early and rapid reduction of HBV DNA levels could suppress hepatocellular necrosis and cytokine release, which slows or reverses the progression of the disease [56]. Studies have indicated that nucleoside analogues could significantly reduce HBV DNA levels in patients with HBV-ACLF, and the reduction in HBV DNA levels within 2 weeks is related to survival improvement [56, 57]. All patients with HBV infection at presentation should be treated with nucleoside analogues immediately. Potent antiviral drugs, such as tenofovir, tenofovir alafenamide or entecavir, should be used [9].

#### Infection

The prevalence of infection in ACLF patients is approximately 50% and rises to more than 70% in ACLF-3 [58]. Bacterial infections are more common than fungal infections [58]. Studies have suggested that bacterial infections are associated with a worse clinical course and high short-term mortality in patients with ACLF [10, 58, 59]. Early identification of bacterial infections is important for the management of ACLF. A comprehensive examination for signs and symptoms of infection, including cytological and microbiological examinations of blood and ascites and imaging examinations of suspected sites, should be systematically performed in all patients at admission before starting antimicrobial therapy. An effective antibiotic treatment is strongly associated with an improvement in survival. Thus, empirical high-dose broad-spectrum antibiotic therapy, which relies on the organism isolated, suspected site of infection and local epidemiological patterns, should be started in ACLF patients with possible infection [60, 61]. Empirical antifungal therapy in patients without risk factors for invasive fungal infections is not recommended [52].

#### Alcohol-related hepatitis

Alcohol-related hepatitis is a major precipitating event of ACLF worldwide. Corticosteroids are the first-line treatment for severe alcoholic hepatitis. The response to corticosteroids can be assessed by dynamically calculating the Lille score, and the probability of response is negatively correlated with the number of OFs at baseline (52% for 1 OF, 42% for 2 OFs and 8% for 3 OFs) [53, 62–64]. Moreover, patients who responded to the treatment exhibited a higher short-term survival rate [64, 65]. However, considering the high risk of bacterial infections in patients with ALD-ACLF [52] and HBV reactivation in patients with HBV-ACLF [66], the benefit of corticosteroids should be carefully evaluated before use [55].

#### Acute variceal haemorrhage

For patients with cirrhosis, acute variceal haemorrhage is a common precipitating event. Standard medical treatment for this life-threatening precipitant is the combination of a vasoconstrictor (including terlipressin, somatostatin or analogues such as octreotide, maintained for 2–5 days) and endoscopic therapy (endoscopic variceal ligation or endoscopic sclerotherapy) with antibiotic prophylactic therapy [53, 67]. In addition, preemptive transjugular intrahepatic portosystemic shunts might also improve the survival of ACLF patients with acute variceal haemorrhage; however, further studies are still needed to validate their role in outcomes in patients with ACLF [68].

### Organ failure support

The artificial liver support system (ALSS) has been recognized as a promising alternative therapy to LT in patients with ACLF by replacement of the dysfunctional liver since it can remove toxins, modulate haemodynamics, clear cytokines, and improve metabolism and consciousness [69–73]. To date, various ALSSs have been tried as treatments for ACLF [73–75]. Albumin dialysis with the molecular adsorbent recirculation system and fractionated plasma separation and adsorption, the most commonly used ALSS models for the treatment of ALD-ACLF in Europe, have shown beneficial effects for improving hepatic and renal functions [76–79]. Recently, a retrospective study and a meta-analysis showed that the use of an ALSS could improve short-term survival (14 and 28 days) in patients with ACLF and multiple OFs [80, 81]. However, two large randomized clinical trials demonstrated no improvement in short-term survival in ACLF patients treated with albumin dialysis compared with standard medical therapy [76, 78]. Other studies on plasma exchange, haemofiltration, and haemoperfusion have shown benefits for the short-term survival of HBV-ACLF and have considered these models as bridges to LT [70, 73, 82]. The clinical utility of these systems for survival benefit was different among studies, and further prospective randomized clinical trials are still needed.

Principles of treatment for extrahepatic OFs of ACLF are summarized in Table 3. Recommendations are based on current clinical guidelines and recent reviews on the management of critically ill patients with or without cirrhosis [53, 55, 83, 84].

**Table 3:** Principles of treatment for extrahepatic organ failures of ACLF

Type of organ failure	What should be done	What should be avoided
Coagulation	Perform the test of blood cell count and coagulation status; Administer fibrinogen and/or platelets in patients with hypofibrinogenemia (< 1 g/L) and/or thrombocytopenia (< 20 × 10^9^/L) with invasive procedures; Prophylaxis for deep-vein thrombosis in patients without severe coagulopathy	Avoid correction of INR with fresh frozen plasma for patients without bleeding or a planned procedure
Kidneys	Assess the severity of acute kidney injury (AKI) with modified KDIGO criteria of the International Club of Ascites; 20% albumin (1 g/kg for 48 h) for patients with AKI stage 2–3; For patients with type-1 hepatorenal syndrome: 20% albumin (1 g/kg for 48 h and then 20–40 g/day) + terlipressin (2 mg/24 h) or norepinephrine (0.5 mg/hour, when terlipressin is not available); RRT serves as a bridge to LT	Avoid nephrotoxic drugs, e.g., NSAIDs; Avoid unnecessary RRT or early initiation of RRT
Respiration	Assess respiratory status (calculating the PaO2/FiO2 or SpO2/FiO2 and performing imaging examination); Oxygen inhalation and lung protective ventilation strategy; Endotracheal intubation for patients with West-Heaven grade 3–4 HE to facilitate airway management, prevent aspiration, and control ventilation; PPIs are suggested to be used in patients on a ventilator	Avoid delay in intubation even if with normal blood oxygen level
Circulation	Assess haemodynamic state at admission; Maintain mean arterial pressure > 65 mmHg; Norepinephrine is the first choice of vasopressor, epinephrine and terlipressin serve as additional agents; Administer crystalloids and 5% albumin as resuscitation fluid; Administer 20% albumin for patients with spontaneous bacterial peritonitis, large volume paracentesis or AKI	Avoid using starches formulations; Limit saline solutions in patients with ascites or anasarca
Brain	Evaluation of the mental status, care of the airway, treatment of the precipitating factors, and empiric HE therapy should be performed simultaneously; Use lactulose and enemas to clear the bowel; Use short-acting sedative agents	Avoid deep sedation; Avoid using benzodiazepines; Ventilation in patients without altered mental status should not be considered as brain failure

Recommendations are based on current clinical guidelines and recent reviews on the management of critically ill patients with or without cirrhosis. *ACLF* acute-on-chronic liver failure; *AKI* acute kidney injury; *HE* hepatic encephalopathy; *INR* international normalized ratio; *KDIGO* kidney disease improving global outcomes; *LT* liver transplantation; *NSAIDs* nonsteroidal anti-inflammatory drugs; *PPIs* proton pump inhibitors; *RRT* renal replacement therapy

### Liver transplantation

LT is an effective therapy for ACLF. The results of an international collaborative study in Europe showed that the 1-year post-LT survival was 81% in 234 ACLF patients, providing clear evidence of a survival benefit from LT [85]. Data from other studies based on multicentre cohorts or national registries also confirmed that LT could markedly improve survival in selected patients, even in those with ACLF-3, with a 1-year survival over 80% [86, 87]. However, the prioritization for LT in these specific populations remains unclear. Current organ allocation around the world is based on a prognostic model, called the MELD score, although studies have shown that the MELD score is not sensitive or accurate enough to predict survival in patients with ACLF since the impact of extrahepatic OF is not reflected in the score [88, 89]. The criteria and prognostic scores, which were developed specifically for patients with ACLF, such as the COSSH-ACLF score and CLIF-C ACLF score, should be used to assess the prognosis and to determine the prioritization and ideal timing for LT in patients with ACLF [6, 13, 85, 90, 91]. Researchers from Europe have proposed the worldwide adoption of new organ allocation policies for patients with ACLF and have designed a global study, referred to as the CHANCE study, to solve these issues [92]. In addition, dynamic assessment of the disease severity after hospitalization may be helpful to optimize patients for LT listing and transplantation. The CANONIC study showed that the high risk of early death in patients with ACLF-2 or ACLF-3 made it necessary to consider LT, and the futility of LT may be considered for patients with 4 or more OFs or a CLIC-C ACLF score > 64 (at days 3–7) if they have other contraindications for LT [90]. The COSSH study currently also assessed the outcomes of patients with HBV-ACLF who underwent LT and proposed a net survival benefit-based priority for LT of HBV-ACLF to decrease the risk of futile LT. The COSSH-ACLF II score identified the risk of death on the waitlist and accurately predicted post-LT mortality and survival benefit for HBV-ACLF. Patients with a COSSH-ACLF II score of 7–10 derived a higher net survival benefit from LT [93].

## Future perspectives

Despite improvements in understanding of how patients with ACLF might progress from underlying chronic liver disease to death, controversies in many fields still exist and require further scientific exploration.

With the increasing prevalence of risk factors for the development of chronic liver disease, such as alcohol addiction, drug abuse and obesity, ACLF is expected to become more prevalent in the coming years [1]. However, given regional limitations, any definitions of ACLF now proposed may be considered only transitional definitions, both in the East and in the West. The multiple definitions have also accounted for substantial confusion among multidisciplinary teams caring for ACLF patients. A uniform definition of ACLF applicable in all parts of the world should be based on global, prospectively collected and validated data, which can provide a sufficient theoretical basis for standardized clinical management. Patients with chronic liver disease (with or without cirrhosis), with any type of precipitating event (intrahepatic or extrahepatic), and extrahepatic OFs should be included for data collection to ultimately arrive at a comprehensive definition of ACLF. More efforts are also required to develop and validate more accurate prognostic scores that can accommodate various aetiologies and phenotypes around the world. In addition, the unmet medical needs of diagnosing patients who have a high risk of progression to ACLF (pre-ACLF) at admission are eagerly awaited to be addressed in future studies. Using advanced approaches such as transcriptomics, proteomics and metabolomics, biomarkers including inflammatory markers, cell-death markers and functional characteristics of immune cells would also help accurately identify pre-ACLF patients to refine clinical prognostic models and provide novel therapeutic targets [94].

To delve deeper into the pathogenesis of ACLF, future work should not only decipher the respective contributions of each immune cell type and their interactions and increase our understanding of dynamic changes in inflammatory, immune and metabolic status during the natural history of ACLF but also generate tractable therapeutic targets through recent advances in single-cell genomics technologies and the rapidly evolving fields of spatial transcriptomic and proteomic profiling [95, 96]. Reliable ACLF animal models are needed to uncover detailed features of the disease pathogenesis. Inflammation-induced ACLF models, in which chronic injury is induced by carbon tetrachloride, bile duct ligation or porcine serum and acute injury is induced by D-galactosamine/lipopolysaccharide, have a high short-term mortality rate after acute insult and rarely lead to the development of portal hypertension, ascites, and multiple OFs [97–102]. Currently, a bacteria-induced ACLF model has been developed using *Klebsiella pneumoniae*, which provided the survival window to understand the pathophysiology of liver failure in response to infection [103]. A porcine serum-induced liver cirrhosis-based ACLF animal model verified immune-metabolism disorder as the core-axis mechanism in the clinical pathophysiology, which positively contributed to the interpretation of disease pathogenesis [104]. Nevertheless, establishing an HBV-based ACLF model is an arduous task because of the extremely narrow host range of HBV, which mostly infects humans. The application of sophisticated technologies has led to the generation of dual humanized transgenic mice that are susceptible to chronic HBV infection and ultimately generate liver cirrhosis [105]. This model could serve as a potential resource for HBV-ACLF modeling with a suitable secondary trigger.

Several interventions targeting immune regulation and metabolic balance are receiving attention as potential treatments for ACLF. Glucocorticoids can suppress excessive immune responses and have been tried in the treatment of ACLF. However, due to the side effects of glucocorticoids and the rapid changes in the immune status of ACLF patients, only some ACLF patients can benefit from glucocorticoids. Therefore, it is difficult to determine the optimal timing for glucocorticoids in patients with ACLF. Granulocyte-colony stimulating factor (G-CSF), which can induce the mobilization of bone mesenchymal stem cells, has been studied to reduce the short-term mortality of ACLF [106]. A meta-analysis demonstrated that G-CSF significantly reduced short-term mortality and the incidence of complications [107]. However, this result was not validated in a recent multicentre randomized clinical trial, which enrolled 176 patients with ACLF from 18 European centres; the interim analysis showed no benefit of G-CSF on 90- or 360-day LT-free survival, overall survival or disease severity scores [108]. Based on current data, the use of G-CSF is debatable. G-CSF may be beneficial in early stages of ACLF before the onset of sepsis and extrahepatic OFs [109] but may not be recommended as a part of routine management for patients in late stages of ACLF [52]. Stem cell therapies, including human allogeneic liver-derived progenitor cell, mesenchymal stem cell (MSC) or multipotent MSC and bone mesenchymal stem cell therapies, are emerging as alternatives because of the shortage of liver donors and the high cost of LT. A clinical phase II study that involved 24 patients suggested that the intravenous infusion of low doses of human allogeneic liver-derived progenitor cells to treat patients with ACLF is safe [110]. Other studies have demonstrated that the transplantation of MSCs improved liver function and short-term survival in HBV-ACLF, and the procedure appeared to be safe during the observational time [111, 112]. However, these studies were preliminary, with small sample sizes and limited follow-up periods. Further studies are required to confirm the safety and assess the efficacy before widespread use in clinical practice.

## Conclusions

ACLF has been recognized as a severe clinical syndrome based on the acute deterioration of chronic liver disease, and it is characterized by organ failure and high short-term mortality. The specific pathophysiology of ACLF remains unclear, but it is likely related to systemic inflammation and immune-metabolism disorder. Current therapeutic management of ACLF relies on supportive therapy for organ failure and liver transplantation. ACLF is still a major challenge in the field of hepatology, and future studies are needed to clarify its pathogenesis and achieve precise management through spatiotemporal multi-omics.

## Data Availability

Not applicable.

## Abbreviations

ACLF: Acute-on-chronic liver failure
AARC: Asian Pacific Association for the Study of the Liver ACLF Research Consortium
ALD: Alcohol-related liver disease
ALSS: Artificial liver support system
APASL: Asian Pacific Association for the Study of the Liver
AUROC: Area under the receiver operating characteristic curve
CANONIC: Chronic liver failure (CLIF) consortium acute-on-chronic liver failure in cirrhosis
C-index: Concordance index
COSSH: Chinese Group on the Study of Severe Hepatitis B
DAMPs: Damage-associated molecular patterns
EASL-CLIF: European Association for the Study of the Liver-Chronic Liver Failure
G-CSF: Granulocyte-colony stimulating factor
HBV: Hepatitis B virus
HE: Hepatic encephalopathy
INR: International normalized ratio
IL: Interleukin
LT: Liver transplantation
MELD: Model for end-stage liver disease
MSC: Mesenchymal stromal cell
NACSELD: North American Consortium for the Study of End-stage Liver Disease
OFs: Organ failures
PRRs: Pattern-recognition receptors
PAMPs: Pathogen-associated molecular patterns
SOFA: Sequential organ failure assessment
TB: Total bilirubin
WBC: White blood cell
